# Assessment of Oral and Dental Health Status Among Turkish Individuals with Coronary Artery Disease: A Cross-Sectional Study

**DOI:** 10.3390/healthcare14213646

**Published:** 2026-10-28

**Authors:** Amine Terzi, Hülya Keskin

**Affiliations:** 1 Department of Internal Medicine Nursing, Faculty of Health Sciences, Artvin Çoruh University, 08100 Artvin, Turkey; 2 Department of Audiology, Faculty of Health Sciences, Mardin Artuklu University, 47100 Mardin, Turkey; hulyakeskin@artuklu.edu.tr

**Keywords:** coronary artery disease, cross-sectional studies, health knowledge, attitudes, practice, nursing care, oral health, oral hygiene

## Abstract

Background: Poor oral health is associated with cardiovascular disease, but nursing-focused evidence in coronary artery disease (CAD) is limited in Turkey. This study assessed oral and dental health status among Turkish individuals with CAD and its relationship with sociodemographic, clinical, and behavioural factors. Methods: In this cross-sectional study, 394 patients with CAD were consecutively enrolled from a cardiology department and outpatient clinic in Mardin, Turkey. Data were collected with two researcher-developed forms and the Turkish Oral Health Assessment Tool (OHAT; 0–16), and analysed with group comparisons, correlations, and an a priori path model with bootstrapped indirect effects. Results: The mean OHAT score was 3.43 ± 2.58, and 86.0% met the tool’s dental referral criterion. Most (91.9%) visited a dentist only when they had a complaint, 21.1% never brushed, and 88.3% reported having no oral hygiene information. Higher OHAT scores were associated with longer diagnosis duration, more comorbidities, lower education, less frequent brushing, complaint-only dental visits, and lack of oral hygiene information. Age and education were indirectly associated with OHAT score through oral care behaviour. Conclusions: Oral health practices were poor. Routine nursing oral health assessment with dental referral may be warranted; the benefit of nurse-led education requires interventional testing.

## 1. Introduction

Coronary artery disease (CAD) remains a leading cause of morbidity and mortality in adults [1]. Beyond the classic risk factors of hypertension, dyslipidaemia, smoking, diabetes and advanced age, oral health has attracted growing attention as a potentially modifiable correlate of cardiovascular disease. Proposed biological pathways include bacteraemia and endothelial invasion by periodontal pathogens, platelet aggregation, and a chronic systemic inflammatory burden (e.g., C-reactive protein, interleukins and fibrinogen) that may contribute to atherogenesis [2,3,4,5].

Evidence synthesised in systematic reviews and meta-analyses indicates that this association is not limited to periodontitis. A meta-analysis of prospective studies reported summary relative risks for incident coronary heart disease ranging from 1.24 (95% CI 1.01–1.51) to 1.34 (95% CI 1.10–1.63) across categories of periodontal disease [6], and a more recent meta-analysis found that the association between periodontal disease and cardiovascular disease was significant in both men and women [7]. A dose–response meta-analysis of prospective cohorts showed that every two additional teeth lost were associated with a 3% increase in coronary heart disease risk [8], and an evidence summary of 22 systematic reviews concluded that periodontitis, dental caries and tooth loss are all associated with cardiovascular disease, although a causal relationship has not been established [9]. Oral hygiene behaviour itself also appears relevant: in a Korean population-based cohort, each additional daily tooth brushing was associated with a 9% lower risk of cardiovascular events and regular professional dental cleaning with a 14% lower risk [10], and in the Scottish Health Survey, participants who rarely or never brushed their teeth had a higher risk of cardiovascular disease events (HR 1.7, 95% CI 1.3–2.3) [11]. Consensus reports have accordingly recommended that health professionals caring for people with cardiovascular disease ask about oral symptoms and provide individualised oral hygiene advice [12,13].

Nevertheless, much of this association may reflect shared risk factors and health behaviours. In a Finnish cohort, oral health indicators no longer predicted coronary deaths after adjustment for education and conventional risk factors [14]; in a cohort of one million Koreans, the association between missing teeth and coronary heart disease disappeared among never-smoking men [15]; and oral health has been proposed as a risk marker rather than a risk factor for cardiovascular disease [16]. Oral health in CAD is therefore best approached as a clinically relevant and modifiable aspect of patient care rather than as an established causal risk factor, and the present study adopts a descriptive and associative framework that does not claim causality.

Most of the available evidence relies on clinical periodontal or dental examinations performed by dental professionals, such as probing depth, attachment loss or tooth counts [17,18]. In routine cardiology settings, comprehensive dental examination is rarely available, whereas nurses are in continuous contact with patients. Assessing general oral health status—the lips, tongue, gums and oral tissues, saliva, natural teeth, dentures, oral cleanliness and dental pain—is distinct from diagnosing periodontal disease, but it is within the scope of nursing assessment and can identify patients who need referral to a dentist. The Oral Health Assessment Tool (OHAT) was developed for this purpose for use by nurses and care staff [19], and its Turkish version has established content validity and inter-rater reliability [20]. Evidence on nurse-administered oral health screening in patients with CAD, however, is scarce.

Oral care behaviours are a plausible link between patients’ sociodemographic characteristics and their oral health. Among patients with CAD in Pakistan, daily interdental cleaning was rare, most patients were unaware of the link between oral and general health, and cardiac care providers seldom offered oral health information [21]; Korean national data indicate that adults at cardiovascular risk need targeted oral health education [18]; and adherence to preventive dental care has been associated with lower healthcare costs in patients with CAD [22]. Because tooth brushing, interdental cleaning and having oral hygiene information are modifiable and amenable to nursing education, examining how they relate both to patient characteristics and to observed oral health status may help identify patients who would benefit most from nursing attention.

In Turkey, evidence on oral health in CAD is limited to a small clinical periodontal study of 70 patients with angiographically confirmed CAD, which reported a high prevalence of periodontitis [17]; data on the general oral health status, oral care behaviours and their correlates in Turkish patients with CAD, assessed from a nursing perspective, are lacking. The aim of this study was therefore to assess oral and dental health status among Turkish individuals with CAD and its relationship with sociodemographic characteristics, comorbidity burden and oral care behaviours. The research question was ‘What is the oral and dental health status of Turkish individuals with CAD, and with which sociodemographic, clinical and behavioural factors is it associated?’ In line with the descriptive, cross-sectional design, no formal hypotheses were tested; the path analysis was an exploratory examination of a conceptual framework specified a priori (Section 2.4).

## 2. Materials and Methods

### 2.1. Design

This was a cross-sectional, descriptive study. Data were collected between 25 September 2023 and 31 January 2024 in the Cardiology Department and Cardiology Outpatient Clinic of Mardin Training and Research Hospital, Mardin, Turkey. This report follows the STROBE (Strengthening the Reporting of Observational Studies in Epidemiology) guideline for cross-sectional studies.

### 2.2. Population, Sample, and Participant Selection

The study population consisted of patients with CAD treated in or presenting to these units during the study period. No sampling method was used; all eligible patients were invited to participate (complete enumeration), and 394 individuals constituted the study sample.

#### 2.2.1. Inclusion Criteria

Participants were included if they (i) were aged 18 years or older; (ii) had been diagnosed with CAD at least one month before data collection; (iii) were being treated in the cardiology ward or attended the cardiology outpatient clinic during the study period; (iv) had the mental and cognitive capacity to respond to the data collection forms; and (v) agreed to participate voluntarily and provided written and verbal informed consent.

#### 2.2.2. Exclusion Criteria

Individuals were excluded if they (i) declined to participate; (ii) had been diagnosed with CAD less than one month before data collection; or (iii) had a cognitive or communication impairment that prevented completion of the face-to-face interview and the oral assessment.

#### 2.2.3. Sample Size

No a priori power analysis was performed, as all eligible patients were to be enrolled; the adequacy of the achieved sample for the path analysis was evaluated in two ways. First, the primary path model had 21 free parameters (9 regression coefficients, 2 residual variances and 10 variances/covariances of the exogenous variables), giving a ratio of approximately 19 participants per estimated parameter, which exceeds the commonly recommended minimum of 10:1 and approaches the more conservative 20:1 ratio [23]. Second, a Monte Carlo simulation (1000 replications at N = 394, with Monte Carlo confidence intervals for the indirect effect) showed that power to detect an indirect effect was 0.95 when both component paths were β = 0.20 and >0.99 when both were β = 0.26, but approximately 0.60 when both were small (β = 0.14), in line with published sample size requirements for mediated effects [24]. The sample was therefore adequate for indirect associations of small-to-medium size but may have been underpowered for very small ones.

### 2.3. Data Collection Instruments

Data were collected in face-to-face interviews using two researcher-developed forms and one standardised instrument.

#### 2.3.1. Individual Descriptive Characteristics Form

This 26-item form covers participants’ sociodemographic characteristics, such as age, sex, marital status, education level, occupation, and perceived income, as well as information related to their disease and treatment (diagnosis duration, accompanying chronic diseases, treatment received, etc.).

#### 2.3.2. Oral and Dental Health Practices Questionnaire

This 12-item form (the last item, on the source of oral hygiene information, was answered only by participants who reported having such information) assesses oral and dental health practices, including frequency of dental visits, frequency and duration of tooth brushing, use of dental floss/toothpicks, and whether participants had information about oral hygiene. Item content was based on the literature, drawing on measures used in studies of oral hygiene behaviours and awareness in patients with CAD [13,18,21]. The form consists of single-item behavioural questions with categorical response options (e.g., tooth-brushing frequency: never, occasionally, once, twice or three times a day; dental floss use: never, occasionally, once a day or twice a day; having information about oral hygiene (‘Do you have information about oral hygiene?’): yes/no). It is not a multi-item psychometric scale and yields no total score; each item was analysed separately, and three items were combined into the composite index described in Section 2.4. No formal expert panel review, content validity index calculation, pilot study or reliability testing was performed before data collection; this is acknowledged as a limitation (Section 4.3).

#### 2.3.3. Oral Health Assessment Tool (OHAT—Turkish Version)

The OHAT was developed by Chalmers et al. for oral health screening of older adults, including people with cognitive impairment, by nurses and care staff rather than dental professionals; the original version showed acceptable intra-rater (r = 0.78) and inter-rater (r = 0.74) reliability for total scores [19]. The Turkish version (OHAT-TR) was adapted by Şahin and Jablonski through forward–backward translation and was evaluated by six experts in nursing and dentistry (content validity indices >0.80); in 100 cognitively intact nursing-home residents aged 65 years or older, inter-rater agreement ranged from κ = 0.54 to 1.00 for the individual categories and was ρ = 0.72 for the total score [20]. The tool comprises 8 categories—lips, tongue, gums/tissue, saliva, natural teeth, dentures, oral cleanliness and dental pain—each scored 0 (healthy), 1 (changes) or 2 (unhealthy), yielding a total score ranging from 0 (very healthy) to 16 (very unhealthy). According to the scoring instructions, a score of 1 or 2 in any category indicates that the person should be examined by a dentist. Neither the original nor the Turkish version defines cut-off points for the total score [19,20]; the total score was therefore analysed as a continuous variable. For descriptive purposes only, we also report (a) the proportion with a total score ≥ 1 (i.e., at least one category scored 1 or 2, the instrument’s referral criterion) and (b) three researcher-defined bands of approximately equal width over the possible score range (0–4, 5–9 and 10–16). These bands are not validated clinical thresholds and were not used in inferential analyses. Because OHAT-TR was validated in nursing-home residents aged 65 years and older, its use in a younger, hospital-based CAD population is an extrapolation; however, the OHAT categories describe directly observable oral conditions whose definitions are not age-specific, and the Turkish validation sample, like ours, included a high proportion of illiterate participants (43%) [20]. Permission to use the form was obtained from the developers by email.

#### 2.3.4. Clinical Oral Assessment Procedure

Oral assessments were performed face-to-face by the researcher (H.K.), who holds a doctoral degree in internal medicine nursing, after the questionnaire interview—at the bedside for inpatients and in an examination room for outpatients. Using disposable gloves, a penlight and a wooden tongue depressor, the assessor inspected the lips, tongue, gums and oral mucosa, saliva, natural teeth, dentures (if present) and oral cleanliness, and assessed dental pain by asking the patient about pain and observing the verbal, behavioural and physical signs listed in the OHAT-TR descriptors. Each category was scored 0, 1 or 2 against the OHAT-TR descriptors, and the scores were summed. Only the total score was entered into the analysis dataset, and inter-rater reliability was not assessed in the present study.

### 2.4. Data Analysis

Data were summarised as mean ± standard deviation (SD), median (interquartile range, IQR) and frequency/percentage. The distribution of the OHAT total score was examined with the Shapiro–Wilk test and skewness/kurtosis values, and homogeneity of variances with the Brown–Forsythe (median-centred Levene) test. Two-group comparisons used Welch’s *t*-test, which does not assume equal variances. Comparisons of more than two groups used one-way ANOVA, with Welch’s ANOVA reported alongside, and significant omnibus results were followed by Games–Howell post hoc comparisons, which are appropriate for unequal group sizes and variances [25]. Because OHAT scores departed from normality and some groups were small, Mann–Whitney U and Kruskal–Wallis tests were performed as robustness checks, and associations with continuous variables were assessed with both Pearson and Spearman correlation coefficients. Education level was recorded in six ordered categories (1 = illiterate, 2 = literate without formal schooling, 3 = primary school, 4 = secondary school, 5 = high school, 6 = college/university); it was analysed as a six-group factor in group comparisons, so that no linear trend was imposed, and as an ordinal variable (via polyserial correlations) in the path analysis. A *p*-value < 0.05 was considered statistically significant.

- Oral care behaviour index: Because oral care behaviour was not measured by a validated scale, an index was constructed a priori from three questionnaire items representing the core components of personal oral self-care recommended for people with cardiovascular disease—tooth brushing, interdental cleaning and being informed about oral hygiene [12,13]: tooth-brushing frequency (0 = never to 4 = three times a day), dental floss use frequency (0 = never to 3 = twice a day) and having oral hygiene information (0 = no, 1 = yes). Each item was converted to a z-score, and the index was calculated as the unweighted mean of the three z-scores (higher values indicating better oral care behaviour); equal weights were used because there was no empirical or theoretical basis for differential weighting. The index is conceptualised as formative—the indicators jointly define the construct rather than reflecting a common latent cause—so high inter-item correlations or internal consistency are not expected [26]; the same rationale applies to the OHAT total score [20]. The indicators were positively and weakly to moderately inter-correlated (Spearman ρ = 0.17–0.27; polychoric r = 0.31–0.54). Because combining distinct behaviours may mask indicator-specific associations, the path analysis was repeated with the individual indicators (see below).
- Path model specification: A conceptual framework was specified before the analyses on the basis of the literature. Sociodemographic and clinical characteristics (age, education level, number of comorbidities and log-transformed diagnosis duration) were expected to be associated with oral care behaviour—older age and lower education with poorer self-care and less access to oral health information [18,21], and a greater comorbidity burden and longer disease duration with competing self-care demands—and oral care behaviour was expected to be associated with OHAT score [10,11]. Direct paths from all four characteristics to OHAT score were also specified, because age, chronic disease burden and disease duration may relate to oral status through non-behavioural routes (e.g., cumulative tooth loss or medication-related changes) [8,13]. All paths of this a priori model were retained and are reported as the primary analysis, irrespective of their statistical significance; because the model is just-identified, global fit indices are not informative and inference focuses on the path coefficients. A simplified (trimmed) model, obtained by removing three non-significant paths (number of comorbidities → oral care behaviour, diagnosis duration → oral care behaviour and age → OHAT score), is presented only as a sensitivity analysis. Diagnosis duration was log-transformed because of marked right skew (skewness = 2.06 before and 0.49 after transformation).
- Estimation: Because education level and the behavioural indicators are ordinal or binary, the models were estimated from a heterogeneous correlation matrix comprising polychoric (ordinal–ordinal), polyserial (ordinal–continuous) and Pearson (continuous–continuous) correlations, each estimated by two-step maximum likelihood [27]; this is the input used by categorical estimators such as diagonally weighted least squares (DWLS/WLSMV) [28]. For a recursive, just-identified path model, the DWLS, unweighted least squares and ML discrepancy functions yield identical point estimates from a given correlation matrix, so standardised coefficients were obtained directly from this matrix. Standard errors, *p*-values and 95% percentile confidence intervals (CIs) for direct, indirect (product-of-coefficients) and total effects were obtained by nonparametric bootstrapping with 2000 resamples, re-estimating the full polychoric/polyserial matrix in each resample. Three sensitivity analyses were performed: (i) a model in which tooth-brushing frequency, dental floss use and having oral hygiene information were entered as three parallel ordinal mediators with correlated residuals (in the path models, dental floss use was dichotomised as never vs. any use, because only one participant reported daily use); (ii) three models each including a single indicator; and (iii) the simplified (trimmed) model. Given the cross-sectional design, the temporal ordering of predictors, behaviour and OHAT score cannot be established; indirect effects are therefore interpreted as exploratory indirect statistical associations, not as evidence of mediation in a causal sense [29]. Analyses were performed in Python 3.12.10 (Python Software Foundation, Beaverton, OR, USA; pandas 3.0.3 (NumFOCUS, Inc., Austin, TX, USA), SciPy 1.18.0 (NumFOCUS, Inc., Austin, TX, USA), statsmodels 0.14.6 (NumFOCUS, Inc., Austin, TX, USA) and semopy v2.3.11 (Peter the Great St. Petersburg Polytechnic University, St. Petersburg, Russia), with custom routines for polychoric/polyserial correlations and bootstrapping).

There were no missing data for any study variable, with the exception of the item on source of oral hygiene information, which applied only to participants who reported having such information (*n* = 46) and was therefore not applicable to the remainder of the sample by design. Body mass index (BMI) was calculated from self-reported weight and height as weight (kg) divided by height squared (m^2^) and classified according to World Health Organization criteria; two implausible height entries (70 cm and 1670 cm) were considered data-entry errors and corrected to 170 cm and 167 cm, respectively, for the BMI calculation only. No subgroup or interaction analyses were performed, as these were outside the scope of the study’s descriptive aim. To reduce selection bias, all eligible patients were invited rather than using a convenience sample, although single-centre recruitment remains a source of selection bias (Section 4.3). Measurement and information biases were reduced by using a standardised instrument (OHAT-TR) and structured, face-to-face data collection.

### 2.5. Ethical Considerations

The study was approved by the Non-Interventional Clinical Research Ethics Committee of Mardin Artuklu University (protocol code 2023/9-7, approved on 18 September 2023) and was conducted in accordance with the Declaration of Helsinki. Written and verbal informed consent was obtained from all participants prior to data collection.

## 3. Results

### 3.1. Sociodemographic Characteristics

The mean age of the 394 participants was 59.8 ± 14.2 years (range: 18–94); 58.4% (*n* = 230) of participants were male and 89.1% (*n* = 351) were married. Regarding education level, 33.0% of participants were illiterate and 27.2% were primary school graduates; only 3.8% were college/university graduates. According to World Health Organization criteria, 60.6% (*n* = 239) of participants were classified as overweight or obese (Table 1).

### 3.2. Disease- and Treatment-Related Characteristics

Of the participants, 63.7% were diagnosed because of chest pain and 36.3% because of a heart attack. Diagnosis duration showed a right-skewed distribution, with a median value of 4 months (mean 36.7 ± 59.3 months); 62.2% of participants had been diagnosed within the past year. The most common accompanying chronic disease was hypertension (52.8%); the mean number of comorbidities was 1.41 ± 1.12 (range: 0–5). Participants had been hospitalised a mean of 2.51 ± 3.49 times because of CAD (Table 2).

### 3.3. Oral Care Practices of Participants

Oral and dental health behaviours were generally inadequate (Table 3): 91.9% of participants visited the dentist only when they had a complaint, 21.1% never brushed their teeth, and 34.3% brushed occasionally; only 2.0% brushed three times a day. Overall, 91.1% never used dental floss, and 88.3% reported having no information about oral hygiene; among the 46 participants who reported having information, the most common sources were family (30.4%) and television (26.1%), and only one each named a nurse or a physician.

### 3.4. Total Oral Health Assessment Tool (OHAT) Score

The mean total OHAT score was 3.43 ± 2.58 (median = 3, IQR = 1–5, range: 0–12). Only 14.0% of participants (*n* = 55) had a total score of 0; the remaining 86.0% (*n* = 339) had at least one OHAT category scored 1 or 2 and thus met the instrument’s criterion for referral to a dentist. In the researcher-defined descriptive bands, 68.8% of participants scored 0–4, 27.9% scored 5–9 and 3.3% scored 10–16 (Table 4); these bands are not validated clinical categories.

### 3.5. Relationship Between OHAT Score and Sociodemographic and Behavioural Variables

The OHAT total score was moderately right-skewed (skewness = 0.74; excess kurtosis = 0.28) and departed from normality (Shapiro–Wilk W = 0.936, *p* < 0.001); variances were homogeneous in all comparisons (Brown–Forsythe *p* > 0.05) except for sex (*p* = 0.023), for which Welch’s t-test was used, as in all two-group comparisons. No statistically significant association was found between OHAT score and sex (t = 0.766; *p* = 0.444) or smoking status (F = 2.355; *p* = 0.096). In contrast, OHAT score differed significantly by education level (F = 6.110; *p* < 0.001), tooth-brushing frequency (F = 13.462; *p* < 0.001), frequency of dental visits (t = 3.779; *p* < 0.001), dental floss use (t = 2.216; *p* = 0.032) and having oral hygiene information (t = 5.138; *p* < 0.001). All of these results were confirmed by Welch’s ANOVA and the non-parametric tests, with the exception of dental floss use, for which the Mann–Whitney U test was not significant (*p* = 0.067); this association should therefore be regarded as not robust (Table 5).

The pattern across education groups was non-linear: the highest (worst) mean was observed in the literate-without-formal-schooling group (4.87 ± 3.09; *n* = 45), followed by the illiterate group (3.64 ± 2.62; *n* = 130); from primary school onward, the mean decreased as education level increased, with college/university graduates having the lowest (best) mean (1.80 ± 2.70; *n* = 15). Games–Howell comparisons showed that the literate-without-formal-schooling group had significantly higher scores than the secondary-school (*p* = 0.039), high-school (*p* < 0.001) and college/university (*p* = 0.012) groups, and that the illiterate group had higher scores than the high-school group (*p* = 0.015); no other pairwise difference was significant, including that between the illiterate and literate-without-formal-schooling groups (*p* = 0.175). Across the six ordered groups, OHAT scores decreased modestly with increasing education (Spearman ρ = −0.184; *p* < 0.001).

OHAT scores decreased stepwise with more frequent tooth brushing, from 4.49 ± 2.26 among participants who never brushed to 0.75 ± 0.71 among those who brushed three times a day. Games–Howell comparisons showed that participants who brushed once a day had lower scores than those who never brushed (*p* < 0.001), that those who brushed twice or three times a day had lower scores than those who never or only occasionally brushed (all *p* < 0.001), and that those who brushed twice a day had lower scores than those who brushed once a day (*p* = 0.009); never and occasional brushers did not differ (*p* = 0.275). Because only eight participants brushed three times a day, the analysis was repeated after merging the twice- and three-times-a-day groups (*n* = 56), with unchanged conclusions (Kruskal–Wallis H = 58.46; *p* < 0.001).

OHAT scores were lower among those who used dental floss (2.60 ± 2.29) than among those who did not (3.51 ± 2.59), and among those who reported having oral hygiene information (1.89 ± 2.10) than among those who had not (3.63 ± 2.57). Frequency of dental visits was dichotomised into visiting only when having a complaint and visiting once a year or more often; OHAT scores were higher (worse) in the complaint-only group (3.54 ± 2.59; *n* = 362) than in the group visiting once a year or more often (2.13 ± 1.98; *n* = 32).

OHAT score showed statistically significant, weak-to-moderate positive correlations with age (r = 0.151; *p* = 0.003), diagnosis duration (r = 0.200; *p* < 0.001) and number of comorbidities (r = 0.229; *p* < 0.001). No significant association was found between BMI and OHAT score (r = 0.015; *p* = 0.766). Spearman correlation coefficients led to the same conclusions, although the correlation with age was weaker (ρ = 0.107; *p* = 0.034) (Table 6).

### 3.6. Path Analysis Findings

**Figure 1:**
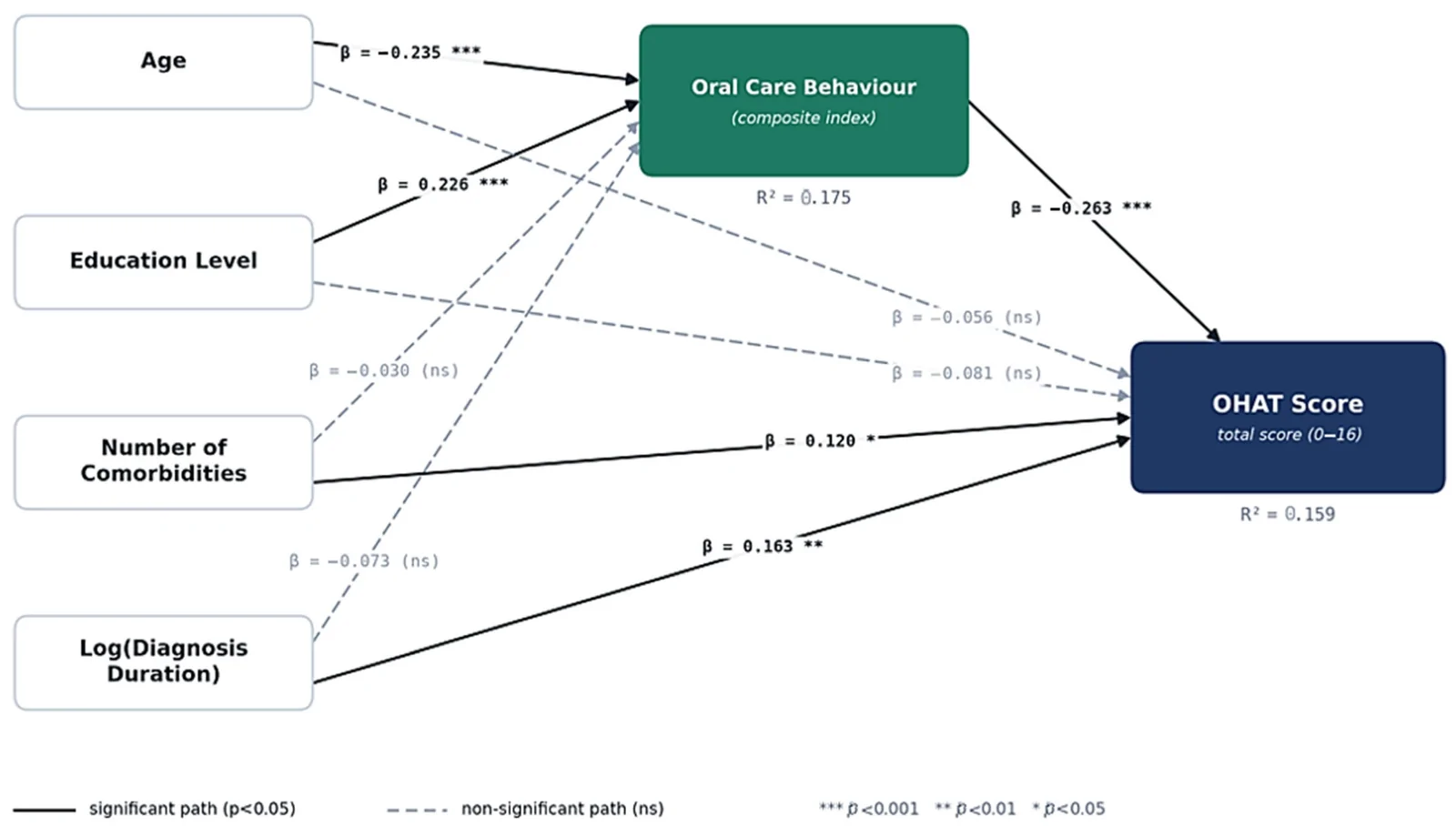
A priori path model (primary analysis) with standardised coefficients estimated from the polychoric/polyserial correlation matrix; significance is based on 2000 bootstrap resamples. Dashed arrows indicate non-significant paths (*p* ≥ 0.05); exogenous covariances and residual terms are omitted for clarity.

Because OHAT is represented in the dataset only as a total score and the other variables were single items, a latent-variable structural equation model could not be constructed; a path analysis of observed variables was therefore performed. In the primary (a priori) model (Table 7, Figure 1), older age (β = −0.235; 95% CI −0.337 to −0.128; *p* < 0.001) and lower education level (β = 0.226; 95% CI 0.120 to 0.327; *p* < 0.001; higher education associated with better behaviour) were associated with poorer oral care behaviour, whereas number of comorbidities (β = −0.030; *p* = 0.564) and log-diagnosis duration (β = −0.073; *p* = 0.109) were not. Better oral care behaviour was associated with a lower (better) OHAT score (β = −0.263; 95% CI −0.347 to −0.165; *p* < 0.001). Number of comorbidities (β = 0.120; *p* = 0.035) and log-diagnosis duration (β = 0.163; *p* = 0.003) were directly associated with higher OHAT scores, whereas the direct paths from age (β = −0.056; *p* = 0.349) and education (β = −0.081; *p* = 0.164) were not significant. The model explained 17.5% of the variance in oral care behaviour and 15.9% of the variance in OHAT score.

Bootstrap analyses showed significant indirect associations of age (β = 0.062; 95% CI 0.028 to 0.102) and education level (β = −0.059; 95% CI −0.093 to −0.029) with OHAT score through oral care behaviour, whereas the indirect associations of number of comorbidities and diagnosis duration were not significant (Table 8). For age, the positive indirect association was accompanied by a small, non-significant negative direct association, so that the total association of age with OHAT score in the multivariable model was close to zero (β = 0.006; 95% CI −0.115 to 0.123); the bivariate correlation between age and OHAT score (r = 0.151) is thus largely accounted for by the correlations of age with oral care behaviour, comorbidity and diagnosis duration. For education, the total association was significant (β = −0.140; 95% CI −0.251 to −0.027), about two-fifths of which was indirect.

#### Sensitivity Analyses

In the indicator-level model with three parallel ordinal mediators, having oral hygiene information (β = −0.443; *p* = 0.003) and tooth-brushing frequency (β = −0.185; *p* = 0.034) were independently associated with OHAT score, whereas dental floss use was not (β = 0.139; *p* = 0.340); dental floss use was moderately correlated with having oral hygiene information (polychoric r = 0.54) and few participants used floss (*n* = 35). The indirect associations of age and education operated mainly through having oral hygiene information, and their total indirect associations remained significant (age: β = 0.109, 95% CI 0.006 to 0.275; education: β = −0.137, 95% CI −0.275 to −0.040). In single-indicator models, tooth brushing carried significant indirect associations for education and diagnosis duration, having oral hygiene information for age and education, and dental floss use for none (Table 9). The simplified (trimmed) model yielded estimates virtually identical to those of the a priori model and fitted the data well (ML: χ^2^(3) = 4.51, *p* = 0.211; CFI = 0.989; TLI = 0.966; RMSEA = 0.036; SRMR = 0.023), and ML estimation based on Pearson correlations also gave nearly identical coefficients (e.g., oral care behaviour → OHAT score β = −0.262). Across all specifications, the direct associations of number of comorbidities and diagnosis duration with OHAT score were positive (β = 0.10–0.23; the comorbidity path did not reach significance in the tooth-brushing-only model, *p* = 0.068).

## 4. Discussion

### 4.1. Assessment of Oral and Dental Health Status and Practices

In this study, the mean OHAT total score of the 394 individuals with CAD was 3.43 ± 2.58 (possible range 0–16). Although the mean score was relatively low, 86.0% of participants had changes in at least one OHAT category and thus met the instrument’s own criterion for dental referral, whereas 31.2% scored 5 or more in the researcher-defined bands. Because these bands are not validated thresholds, the referral criterion and the continuous score provide the more defensible summary. These findings are directionally consistent with the Turkish study by Tüter et al. [17], in which 70% of the 70 patients with CAD had periodontitis and 18.5% had gingivitis on clinical periodontal examination. However, OHAT records observable changes in oral tissues, teeth, dentures and cleanliness and cannot diagnose periodontitis; our results describe general oral health status and should not be compared numerically with periodontal prevalence estimates from studies using clinical periodontal indices [17,18,30].

The findings regarding oral care behaviours point to a marked lack of knowledge and practice in the sample: 91.9% of participants visited the dentist only when they had a complaint, 21.1% never brushed their teeth, 91.1% did not use dental floss, and 88.3% reported having no information about oral hygiene. This pattern resembles that among 270 patients with CAD in Pakistan, of whom 80.7% brushed only once a day, only 5.9% used interdental cleaning aids daily, and 94.1% believed there was no relationship between oral health and general body health [21]. Both studies suggest that the oral health behaviours of individuals with CAD fall short of recommendations and that patients are not adequately informed about oral health during cardiac care, as Parveen et al. also emphasised that cardiac care providers mostly do not provide patients with information on this topic [21].

Studies using comprehensive dental examinations have reported that, compared with controls, patients with CAD have worse plaque and periodontal indices and more missing teeth [31,32,33,34,35,36], and observational studies have linked periodontal disease, tooth loss and edentulism with coronary, cardiovascular and peripheral arterial outcomes [16,37,38,39,40,41]. Our finding that more than four-fifths of participants had at least one OHAT category with changes is consistent with this picture of poor oral health among people with cardiovascular disease. However, because there was no non-CAD comparison group, any CAD-specific interpretation rests on these external comparisons.

Sociodemographic correlates of oral health within populations already diagnosed with CAD have received limited attention, and the present study adds to this literature. The finding that OHAT score increased with increasing number of comorbidities and diagnosis duration (r = 0.229 and r = 0.200, respectively) is consistent with the literature on chronic disease burden and oral health: a European consensus report highlights the bidirectional relationship between periodontal disease and diabetes and their shared inflammatory mechanisms [13], and another study showed that oral health-related quality of life is significantly lower in individuals with cardiovascular disease than in healthy individuals [30].

The education findings warrant cautious interpretation. OHAT scores were lowest among high-school and university graduates, but the relationship was not strictly linear: participants who were literate without formal schooling had the highest scores, higher even than illiterate participants. This group was small (*n* = 45), and its difference from the illiterate group was not significant in post hoc comparisons (*p* = 0.175), so the deviation may reflect sampling variability rather than a true pattern. Among the behavioural indicators, having oral hygiene information showed the strongest association with OHAT score, followed by tooth-brushing frequency, whereas dental floss use was not independently associated with OHAT score and its bivariate association was not robust to non-parametric testing; this probably reflects the very low prevalence of flossing (8.9%) and its overlap with having oral hygiene information. Only 2 of the 46 participants who reported having oral hygiene information named a nurse or physician as the source, which suggests that oral health education is largely missing from cardiac care in this setting.

The absence of a significant association between smoking status and OHAT score (F = 2.355; *p* = 0.096) is noteworthy, as smoking is frequently emphasised in the literature as a shared risk factor for both periodontal disease and CAD [37,39]. However, most such studies compare individuals with and without CAD, whereas all our participants had CAD, which may have reduced the discriminating effect of smoking. In a Korean cohort of one million people, the association between tooth loss and coronary heart disease in men disappeared among never smokers, suggesting that smoking may be a strong confounder in the oral health–cardiovascular disease relationship [15].

### 4.2. Interpretation of Path Analysis Findings

The path analysis suggests that age and education were associated with OHAT score partly through oral care behaviour: older and less educated participants reported poorer oral care behaviour, and poorer behaviour was associated with higher OHAT scores. For education, about two-fifths of the total association was indirect, the remainder being a non-significant direct association. The indirect associations of age and education remained significant when the three behavioural indicators were entered as parallel mediators and in the trimmed model, but they were not uniform across indicators: in single-indicator models, both operated through having oral hygiene information, only education operated through tooth brushing, and neither operated through dental floss use. These associations are exploratory and do not demonstrate mediation. Because all variables were measured at the same time, the temporal ordering implied by the model cannot be verified; reverse pathways (e.g., poor oral status discouraging tooth brushing) and unmeasured confounders such as socioeconomic resources, manual dexterity or access to dental care could produce the same pattern [29]. For age in particular, the total association with OHAT score in the multivariable model was close to zero, so the indirect association does not indicate that age acts through behaviour in a causal sense. These findings are in line with recommendations that oral hygiene advice on brushing and interdental cleaning be individualised for people with cardiovascular disease [12,13] and with evidence that adults at cardiovascular risk need oral health education [18].

The direct associations of comorbidity burden and diagnosis duration with OHAT score, independent of the behaviours measured, suggest that chronic disease burden is related to oral status through other routes. Possible explanations include medication-related xerostomia in patients with polypharmacy, systemic inflammation shared by chronic conditions, reduced capacity for self-care and cumulative dental damage over a longer disease course. None of these factors were measured in the present study; these explanations are therefore hypotheses for future research rather than findings of the present data.

### 4.3. Strengths and Limitations

The main strengths of this study are the inclusion of all eligible patients presenting during the study period (N = 394), the use of a nurse-administered instrument with a published Turkish adaptation (OHAT-TR), and the use of an a priori path model estimated with methods suited to ordinal data, supported by several sensitivity analyses. Several limitations should be considered. First, the cross-sectional design precludes causal inference, and the indirect associations cannot be interpreted as mediation. Second, because there was no non-CAD comparison group, we cannot determine whether the findings are specific to CAD. Third, OHAT is a screening instrument and cannot replace a comprehensive clinical periodontal examination, and no radiographic assessment was performed; subclinical periodontal disease and other conditions detectable only by clinical or radiographic examination, such as those identified on panoramic radiographs [42,43], may therefore have been under-detected. Fourth, OHAT-TR was validated in cognitively intact nursing-home residents aged 65 years or older, and its validity in younger, hospital-based adults with CAD has not been established. Inter-rater reliability was not assessed in this study; only total scores were available, so category-level analyses were not possible, and the OHAT has no validated total-score cut-off points, so the score bands used here are descriptive only. Fifth, the Oral and Dental Health Practices Questionnaire was developed by the researchers without formal expert review, pilot testing or reliability testing, and its items are self-reported and therefore subject to recall and social desirability bias; the oral care behaviour index constructed from these items is a formative composite rather than a validated scale. Sixth, no a priori sample size calculation was performed; the post hoc evaluation indicated adequate power for small-to-medium but not for very small indirect associations. Finally, although complete enumeration avoided convenience sampling, recruitment from a single hospital in south-eastern Turkey may have introduced selection bias, and the findings should be generalised with caution.

### 4.4. Recommendations for Further Research

Future studies should include a comparison group without CAD; combine OHAT with comprehensive clinical and, where appropriate, radiographic dental examinations; record OHAT category scores and assess inter-rater reliability; and use validated measures of oral health behaviour and knowledge. Longitudinal designs are needed to establish the temporal ordering of sociodemographic factors, oral care behaviour and oral health status. The present study did not test any intervention; the potential benefits of structured, nurse-led oral health education for patients with CAD therefore remain hypothetical and should be evaluated in interventional (experimental or quasi-experimental) studies.

## 5. Conclusions

This cross-sectional study showed that oral and dental health knowledge and practices were generally poor among Turkish individuals with coronary artery disease (CAD), and that 86.0% of participants had at least one Oral Health Assessment Tool (OHAT) category indicating the need for dental referral. Higher OHAT scores were associated with longer diagnosis duration, a greater number of comorbidities, lower education level, less frequent tooth brushing, visiting the dentist only when having a complaint and not having oral hygiene information, and, in bivariate analysis, with older age. Exploratory path analysis indicated that age and education had significant indirect associations with OHAT score through oral care behaviour (about two-fifths of the total association for education, whereas the total association for age was close to zero), and that comorbidity burden and diagnosis duration were directly associated with OHAT score; these are statistical associations and do not establish causal pathways. Because no comparison group without CAD was included, it cannot be concluded that these findings are specific to CAD. The results suggest that routine nursing assessment of oral health and oral care behaviours—for example, with OHAT—and referral to dental services where indicated may be worthwhile in cardiac care, particularly for older and less educated patients. Whether nurse-led oral hygiene education improves oral health outcomes in this population was not tested here and requires interventional research.

## Figures and Tables

**Table 1:** Sociodemographic characteristics of participants (N = 394).

Variable	*n*	%
Age (years), mean ± SD (min–max)	59.8 ± 14.2 (18–94)	
Sex		
Female	164	41.6
Male	230	58.4
Marital status		
Married	351	89.1
Single	43	10.9
Education level		
Illiterate	130	33.0
Literate (no formal schooling)	45	11.4
Primary school	107	27.2
Secondary school	46	11.7
High school	51	12.9
College/university	15	3.8
Occupation		
Not working	59	15.0
Homemaker	133	33.8
Civil servant	15	3.8
Worker/labourer	16	4.1
Self-employed	38	9.6
Retired	63	16.0
Student	3	0.8
Other	67	17.0
Perceived income status		
Income higher than expenses	27	6.9
Income equal to expenses	236	59.9
Income lower than expenses	131	33.2
Place of residence		
City (province centre)	108	27.4
District	189	48.0
Town	3	0.8
Village	94	23.9
Smoking status		
Current smoker	109	27.7
Never smoker	212	53.8
Former smoker	73	18.5
Alcohol use status		
Current user	17	4.3
Never user	374	94.9
Former user	3	0.8
BMI classification (kg/m^2^)		
Underweight (<18.5)	4	1.0
Normal (18.5–24.9)	151	38.3
Overweight (25.0–29.9)	149	37.8
Obese (≥30.0)	90	22.8

**Table 2:** Disease- and treatment-related characteristics (N = 394). The percentage column shows the proportion of ‘yes’ responses; the subcategory rows reflect the distribution of the relevant variable.

Variable	*n*	%
Reason for diagnosis		
Chest pain	251	63.7
Heart attack	143	36.3
Diagnosis duration (categorical)		
≤1 year (≤12 months)	245	62.2
>1 to 5 years (13–60 months)	67	17.0
>5 to 15 years (61–180 months)	70	17.8
>15 years (>180 months)	12	3.0
Presence of accompanying chronic disease	295	74.9
Diabetes mellitus	156	39.6
Hypertension	208	52.8
Heart failure	55	14.0
Chronic renal failure	29	7.4
COPD	34	8.6
Other chronic disease	72	18.3
Hospitalisation due to disease	371	94.2
Interventional/medical treatment received		
Angiography	156	39.6
Angiography + stent	191	48.5
Bypass surgery	15	3.8
Medication only	32	8.1
Family history of heart disease	201	51.0
Family/close-circle loss due to heart attack	143	36.3
Use of continuous medication	295	74.9
Regular medication adherence	275	69.8
Number of comorbidities, mean ± SD (min–max)	1.41 ± 1.12 (0–5)	
Number of hospitalisations due to CAD, mean ± SD	2.51 ± 3.49	

**Table 3:** Oral and dental health practices (N = 394).

Variable	*n*	%
Frequency of dental visits		
Only when I have a complaint	362	91.9
Once a year or more often	32	8.1
Tooth-brushing frequency		
Never brush	83	21.1
Occasionally	135	34.3
Once a day	120	30.5
Twice a day	48	12.2
Three times a day	8	2.0
Mean tooth-brushing duration *		
<15 s	75	19.0
15 to <60 s	36	9.1
60 to <90 s	157	39.8
90 to <120 s	68	17.3
120 to <150 s	8	2.0
150–180 s	50	12.7
Tooth-clenching habit	72	18.3
Tooth-grinding habit	60	15.2
Presence of dental braces	11	2.8
Dental floss use		
Do not use	359	91.1
Use (occasionally, *n* = 34; once a day, *n* = 1)	35	8.9
Toothpick use frequency		
Never use	239	60.7
Occasionally	141	35.8
Once a day or more often	14	3.6
Rinsing with plenty of water when unable to brush	361	91.6
Use of products other than water when rinsing	41	10.4
Having information about oral hygiene	46	11.7
Information source (*n* = 46): family	14	30.4
Information source (*n* = 46): television	12	26.1
Information source (*n* = 46): dentist	9	19.6
Information source (*n* = 46): education/programme attended	9	19.6
Information source (*n* = 46): nurse	1	2.2
Information source (*n* = 46): physician	1	2.2

* Mean tooth-brushing duration was self-reported by all participants, including those who reported never or only occasionally brushing. Responses were coded in the dataset as 0–15, 15–60, 60–90, 90–120, 120–150 and 150–180 s; these categories are shown here as half-open intervals.

**Table 4:** Distribution of total OHAT score (mean = 3.43 ± 2.58; median = 3; IQR = 1–5; range = 0–12).

OHAT Total Score (0 = Very Healthy, 16 = Very Unhealthy)	*n*	%
Total score = 0 (all categories healthy)	55	14.0
Total score ≥ 1 (≥1 category scored 1 or 2; dental referral indicated by OHAT)	339	86.0
Researcher-defined descriptive bands *		
0–4 points	271	68.8
5–9 points	110	27.9
10–16 points	13	3.3

* Not validated clinical thresholds; neither the original nor the Turkish OHAT defines total-score cut-off points. The bands are reported for descriptive purposes only.

**Table 5:** Comparison of total OHAT score across sociodemographic and behavioural groups: parametric tests and robustness checks.

Variable	Test Statistic	*p*	Robust Test	*p*
Sex	t = 0.766	0.444	U = 19,783.5	0.404
Smoking status	F = 2.355	0.096	Welch F = 2.060	0.131
			H = 3.694	0.158
Education level	F = 6.110	<0.001	Welch F = 5.484	<0.001
			H = 27.327	<0.001
Tooth-brushing frequency	F = 13.462	<0.001	Welch F = 35.497	<0.001
			H = 59.992	<0.001
Frequency of dental visits	t = 3.779	<0.001	U = 7656.0	0.002
Dental floss use: non-user/user	t = 2.216	0.032	U = 7452.5	0.067
Having oral hygiene information	t = 5.138	<0.001	U = 11,277.5	<0.001

t, Welch’s *t*-test; F, one-way ANOVA; Welch F, Welch’s ANOVA; U, Mann–Whitney U test; H, Kruskal–Wallis test. For two-group comparisons, t and U are given for the first group versus the second (female vs. male; complaint-only vs. regular dental visits; non-users vs. users of dental floss; no information vs. having information), so that positive t values indicate a higher mean OHAT score in the first group. Post hoc comparisons (Games–Howell) are reported in the text.

**Table 6:** Pearson and Spearman correlation coefficients between total OHAT score and continuous variables.

Variable	Pearson r	*p*	Spearman ρ	*p*
Age	0.151	0.003	0.107	0.034
Diagnosis duration (months)	0.200	<0.001	0.215	<0.001
Body mass index	0.015	0.766	−0.004	0.943
Number of comorbidities	0.229	<0.001	0.237	<0.001

**Table 7:** Standardised coefficients of the a priori path model (primary analysis; polychoric/polyserial correlation matrix, 2000 bootstrap resamples).

Path	β	SE	95% CI	*p*
Age → Oral care behaviour	−0.235	0.053	−0.337 to −0.128	<0.001
Education level → Oral care behaviour	0.226	0.053	0.120 to 0.327	<0.001
Number of comorbidities → Oral care behaviour	−0.030	0.051	−0.126 to 0.076	0.564
Log(diagnosis duration) → Oral care behaviour	−0.073	0.046	−0.159 to 0.020	0.109
Oral care behaviour → OHAT score	−0.263	0.046	−0.347 to −0.165	<0.001
Age → OHAT score (direct)	−0.056	0.060	−0.172 to 0.059	0.349
Education level → OHAT score (direct)	−0.081	0.058	−0.195 to 0.030	0.164
Number of comorbidities → OHAT score (direct)	0.120	0.057	0.007 to 0.228	0.035
Log(diagnosis duration) → OHAT score (direct)	0.163	0.054	0.055 to 0.266	0.003

β, standardised coefficient; SE, bootstrap standard error; CI, bootstrap percentile confidence interval. R^2^ = 0.175 (oral care behaviour) and 0.159 (OHAT score). The model is just-identified (df = 0).

**Table 8:** Indirect, direct and total standardised associations with OHAT score in the a priori model (2000 bootstrap resamples, 95% percentile CI).

Predictor	Indirect (Via Oral Care Behaviour)	Direct	Total
Age	0.062 (0.028 to 0.102)	−0.056 (−0.172 to 0.059)	0.006 (−0.115 to 0.123)
Education level	−0.059 (−0.093 to −0.029)	−0.081 (−0.195 to 0.030)	−0.140 (−0.251 to −0.027)
Number of comorbidities	0.008 (−0.021 to 0.034)	0.120 (0.007 to 0.228)	0.127 (0.007 to 0.244)
Log(diagnosis duration)	0.019 (−0.005 to 0.042)	0.163 (0.055 to 0.266)	0.182 (0.070 to 0.289)

**Table 9:** Sensitivity analyses: alternative mediator specifications and model simplification (standardised estimates; 2000 bootstrap resamples).

Model	Mediator → OHAT β (*p*)	Indirect: Age (95% CI)	Indirect: Education (95% CI)	Direct: Comorbidities β (*p*)	Direct: Log Duration β (*p*)
A priori model, composite index (primary)	−0.263 (<0.001)	0.062 (0.028 to 0.102)	−0.059 (−0.093 to −0.029)	0.120 (0.035)	0.163 (0.003)
Three parallel indicators *	Brushing −0.185 (0.034) Floss 0.139 (0.340) Information −0.443 (0.003)	0.109 (0.006 to 0.275)	−0.137 (−0.275 to −0.040)	0.139 (0.026)	0.204 (0.011)
Tooth brushing only	−0.284 (<0.001)	0.018 (−0.014 to 0.055)	−0.048 (−0.088 to −0.015)	0.103 (0.068)	0.127 (0.025)
Dental floss use only	−0.091 (0.458)	0.028 (−0.036 to 0.132)	−0.019 (−0.104 to 0.028)	0.121 (0.047)	0.173 (0.003)
Oral hygiene information only	−0.443 (<0.001)	0.140 (0.056 to 0.277)	−0.135 (−0.276 to −0.050)	0.146 (0.016)	0.226 (<0.001)
Simplified (trimmed) model	−0.251 (<0.001)	0.066 (0.033 to 0.103)	−0.058 (−0.091 to −0.031)	0.110 (0.049)	0.153 (0.004)

* Total indirect association across the three indicators. In single-indicator models, the significant indirect association of diagnosis duration through tooth brushing was β = 0.055 (95% CI 0.022 to 0.092).

## Data Availability

The data supporting the findings of this study are not publicly available in order to protect the confidentiality of participants’ personal data. The data can be obtained from the corresponding author upon reasonable request and within the framework of the relevant ethics committee permissions.

## Abbreviations

The following abbreviations are used in this manuscript:

CAD	Coronary artery disease
OHAT	Oral Health Assessment Tool
BMI	Body mass index
COPD	Chronic obstructive pulmonary disease
SD	Standard deviation
CI	Confidence interval
ML	Maximum likelihood
CFI	Comparative Fit Index
TLI	Tucker–Lewis Index
RMSEA	Root Mean Square Error of Approximation
DWLS	Diagonally weighted least squares
WLSMV	Weighted least squares, mean- and variance-adjusted
IQR	Interquartile range
SE	Standard error
SRMR	Standardised Root Mean Square Residual
OHAT-TR	Oral Health Assessment Tool, Turkish version
ANOVA	Analysis of variance
